# Cyanobacteria in lakes on Yungui Plateau, China are assembled via niche processes driven by water physicochemical property, lake morphology and watershed land-use

**DOI:** 10.1038/srep36357

**Published:** 2016-11-07

**Authors:** Jingqiu Liao, Lei Zhao, Xiaofeng Cao, Jinhua Sun, Zhe Gao, Jie Wang, Dalin Jiang, Hao Fan, Yi Huang

**Affiliations:** 1 State Key Joint Laboratory of Environmental Simulation and Pollution Control, College of Environmental Sciences and Engineering, Peking University, Beijing, 100871, China; 2 Yunnan Key Laboratory of Pollution Process and Management of Plateau Lake – Watershed, Kunming, 650034, China; 3 Chinese Research Academy of Environmental Sciences, Beijing, 100012, China

## Abstract

Plateau lakes are important ecosystems with diverse ecological functions. Cyanobacteria play a key role in plateau lakes as primary producers. However, they are threatening when dense blooms occur. Identifying cyanobacteiral biogeography and the mechanism of assembly processes shaping the distribution of cyanobacteria in plateau lakes is critical for understanding cyanobacterial ecology and applying it to lake management. In the present study, the biogeographic pattern and importance of neutral and niche processes in assembly of cyanobacteria in 21 lakes on Yungui Plateau, China were examined. Results showed that cyanobacteria exhibit unique biogeographic pattern, and most of them have a narrow habitat preference in plateau lakes. They were assembled via niche processes driven by water physicochemical property, lake morphology and watershed land-use, which explained 62.4% of the biological variation. Neutral processes were not at play. Water physicochemical property (key variables - dissolved oxygen, salinity, trophic status and pH) was the most dominant driver shaping its unique biogeographic pattern. Watershed land-use especially urban land, water body and agricultural land also exhibited a strong impact on cyanobacterial distribution, followed by lake morphology. As most of the cyanobacteiral genus detected in these plateau lakes were potential toxin-producers, this study indicated that in order to protect waters from toxic-bloom in the future, reducing nutrient loading and land-use practices are two practical approaches in plateau lake management.

Cyanobacteria are autotrophic prokaryotes that establish themselves in a wide range of terrestrial and aquatic ecosystems due to their high adaptability^1^,^2^,^3^. They play a critical role in sustaining the productivity of ecosystems through photosynthesis and nitrogen fixation, and accumulating phosphorus as cytoplasmic polyphosphate granules^4^,^5^,^6^. It is known that cyanobacteria can rapidly multiply and result in dense blooms in freshwater lakes under eutrophication^3^,^7^,^8^. The biomass accumulated in these blooms can lead to the degradation of aquatic ecosystems by increasing turbidity, reducing water quality and altering aquatic diversity^9^,^10^. Also, certain bloom-forming species can pose a health risk to humans through microcystin production^11^,^12^,^13^. Therefore, studying the biogeographic pattern of cyanobacteria and unraveling their drivers are critical for offering effective strategies for eutrophication control, and minimizing the risk of toxic-bloom formation.

So far, two schools of thought have provided a conceptual framework for explaining microbial biogeography. The first one – niche-based mechanism - suggests that deterministic drivers such as environmental filtering and biotic interactions determine species distribution, while the second one - neutral mechanism – asserts that the biogeographic pattern is simply influenced by neutral factors e.g. ecological drift, dispersal and speciation^14^. Even though a plethora of studies have made efforts to unveil the ecological drivers of cyanobacterial distribution^3^,^8^,^15^,^16^,^17^,^18^,^19^,^20^,^21^,^22^,^23^, at least two issues in our knowledge still remain. Firstly, in many studies only physicochemical factors were considered when studying the influence of niche-based driver on the cyanobacterial distribution^22^,^23^. However, other factors, such as lake hydro-morphology and agricultural land use are also thought to influence the biogeographic pattern^24^,^25^. Secondly, few studies have examined the importance of neutral drivers on the cyanobacterial distribution^26^, especially cyanbacteria in freshwater lakes with high altitude. These issues may result in biased conclusions about the assembly mechanism of cyanobacterial taxa. Thus, it is necessary to study the diversity of cyanobacteria in plateau lakes and clarify the influence of both deterministic driver and neutral driver on the biogeographic pattern of cyanobacteria.

Lakes on the Yungui Plateau, located in southwest of China, possess a wide range of environmental and geographic gradients. Their natural and anthropogenic conditions and geographic locations are ideal for investigating the effect of environmental and neutral factors on the distribution of cyanobacteria. Meanwhile, these lakes serve as important ecosystems for providing numerous habitats for organisms and drinking water sources for local residence after desalination^27^. However, with the growth of population and development of economy in the local area, these lakes are facing plenty of anthropogenic stressors, which cause the shortage of water resource and the deterioration of ecosystem^28^,^29^,^30^,^31^. Studying the distribution of cyanobacteria and the drivers of diversity will benefit the conservation and management of these lakes.

In the present study, the diversity of cyanobacteria that inhabited 21 lakes located on Yungui Plateau, China were analyzed and the quantitative importance of niche and neutral processes on its biogeographic pattern were explored, respectively. The aims of this study were to (1) describe the diversity and distribution of cyanobacteria in multiple plateau lakes, (2) clarify the assembly mechanism for cyanobacterial taxa, (3) quantify the importance of deterministic and neutral drivers shaping the biogeography of cyanobacteria. This study will facilitate current understanding of the diversity and ecology of cyanobacteria, and the limnology and microbiology of plateau lakes. In addition, it will provide critical information for effective management of plateau lakes, such as eutrophication control and toxic-bloom warning.

## Materials and Methods

### Site and sampling

**Figure 1:**
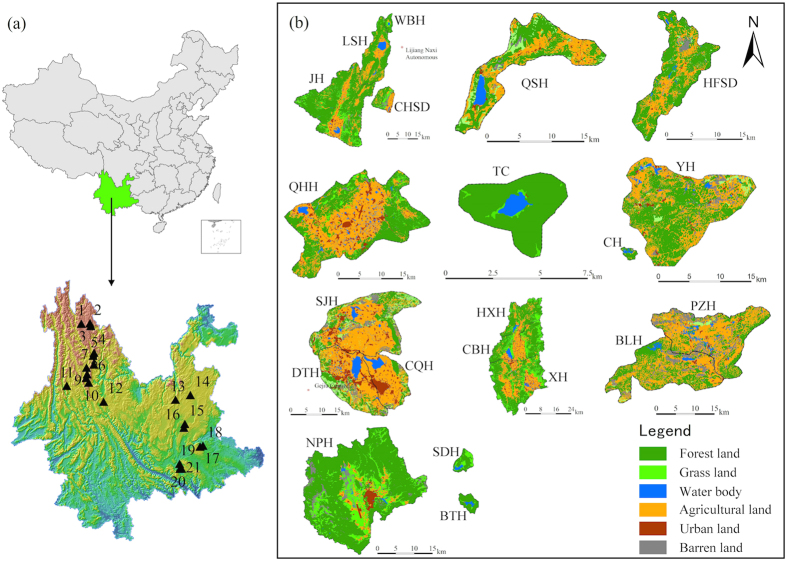
Map of (**a**) 21 sampling lakes located on Yungui Plateau, China and (**b**) the watershed land-use of each lake (ArcGIS 10.1, http://www.esri.com/software/arcgis/arcgis-for-desktop). The code for the lakes is: 1 = Napahai (NPH); 2 = Shuduhai (SDH); 3 = Bitahai (BTH); 4 = Wenhai (WBH); 5 = Lashihai (LSH); 6 = Caihaishidi (CHSD); 7 = Jianhu (JH); 8 = Haixihai (HXH); 9 = Cibihai (CBH); 10 = Xihu (XH); 11 = Tianchi (TC); 12 = Qinghuahu (QHH); 13 = Qingshuihai (QSH); 14 = Haifengshidi (HFSD); 15 = Yuehu (YH); 16 = Changhu (CH); 17- Bailonghe (BLH); 18- Puzhehei (PZH); 19- Sanjiaohai (SJH); 20-Datunhai (DTH); 21-Changqiaohu (CQH).

Datunhai (DTH), Yuehu (YH), Bailonghe (BLH), Qinghuahu (QHH), Changqiaohu (CQH), Changhu (CH), Xihu (XH), Napahai (NPH), Caihaishidi (CHSD), Lashihai (LSH), Haifengshidi (HFSD), Puzhehei (PZH), Sanjiaohai (SJH), Shuduhai (SDH), Wenhai (WBH), Jianhu (JH), Tianchi (TC), Cibihai (CBH), Bitahai (BTH), Haixihai (HXH), and Qingshuihai (QSH) are 21 typical freshwater lakes located on Yungui Plateau, China (latitude from 99.28°E to 104.09°E, longitude from 23.43°N to 27.91°N) (Fig. 1)^14^. These lakes share the characteristics of high altitude locations (above 1200 meters), subtropical plateau monsoon climate, an average annual rainfall of ~1000 mm, low temperature, and high vulnerability to human disturbance^28^,^31^. Water samples were collected from each lake between August and September 2013 with a Ruttner water sampler (Hydro-Bios, Germany, 1,000 mL). The sampling depth was chosen according to Wilhelm *et al.*^23^ and Dadheech *et al.*^2^ as about 15 centimeters below the surface. Subsamples with equal volume were taken from four locations within each lake. Then they were pooled in order to remove temporal and within-lake spatial variability, and to establish a broad and accurate overview of the species^2^,^20^. Samples were immediately placed on ice and returned to the lab for processing. Water samples were pumped through 0.22 μm Sterivex-GP filters (Millipore). The filters were kept at −80 °C until the DNA extraction.

### Environmental variables

A number of variables of water physicochemical property, lake morphology and watershed land-use were included as factors potentially influencing the biologic variation of cyanobacteria (Table S1)^14^. The measured water physicochemical variables were Chlorophyll-a (Chl-a), ammonium-nitrogen (NH_4_^+^-N), nitrate-nitrogen (NO_3_-N), total Kjeldahl nitrogen (TKN), total nitrogen (TN), soluble reactive phosphorus (SRP), total phosphorus (TP), Chemiluminescence detection of permanganate index (CODmn, a proxy for organic pollution), salinity, pH, dissolved oxygen (DO), temperature and transparency (SD). Lake morphological variable includes lake area. Watershed land-use variables were proportion of agricultural land, forestland, grassland, water body, urban land and barren land within each watershed. Chl-a was measured by spectrophotometric method using a Shimadzu UV-1601 spectrophotometer^32^. NH_4_^+^-N, TKN, TN, SRP and TP were determined by USEPA (1983)^33^. NO_3_-N was measured following standard methods^34^. CODmn was determined by the acidic potassium permanganate method^35^. Salinity, pH, DO and temperature were measured *in situ* with a Horiba W-23XD Multi-Parameter Water Quality Meter (Horiba, Japan). SD was measured using Secchi disk according to Davies‐Colley (1988)^36^. Latitude, longitude and altitude were determined by the Global Positioning System (GPS) during field work. Land use classification and coverage of each type at the scale of the entire watershed were obtained in ENVI 4.7 (http://www.harrisgeospatial.com/) using a supervised classification method based on the SPOT 5 satellite images of 2012 with resolution of 2.5 m. ArcGIS 10.1 (http://www.esri.com/software/arcgis/arcgis-for-desktop) was employed to create the map of sampling lakes (Fig. 1) using the vector data of lake and watershed boundary provided by the Yunnan Key Laboratory of Pollution Process and Management of Plateau Lake-Watershed.

### DNA Extraction and PCR amplification

DNA was extracted from water samples using the E.Z.N.A. D5525-01 Water DNA Kit (Omega Bio-tek, Norcross, GA, U.S.) according to standard protocols. The V1-V3 region of the bacterial 16S ribosomal RNA gene was amplified by polymerase chain reaction (PCR) (95 °C for 2 min, followed by 25 cycles at 95 °C for 30 s, 55 °C for 30 s, 72 °C for 30 s and a final extension at 72 °C for 5 min) using primers 27F(5′-AGAGTTTGATCCTGGCTCAG-3′) and 533R(5′-TTACCGCGGCTGCTGGCAC-3′). PCR reactions were performed in a 20 μL mixture containing 4 μL of 5× FastPfu Buffer, 2 μL of 2.5 mM dNTPs, 0.8 μL of each primer (5 μM), 0.4 μL of FastPfu Polymerase, and 10 ng of template DNA.

### 454 pyrosequencing

After purification using the AxyPrep DNA Gel Extraction Kit (Axygen Biosciences, Union City, CA, U.S.) and quantification using QuantiFluor™ -ST (Promega, U.S.), amplicons were pooled prior to pyrosequencing on a Roche 454 GS FLX + Titanium platform (Roche 454 Life Sciences, Branford, CT, U.S.) according to standard protocols^37^. The raw reads were deposited into the NCBI Sequence Read Archive (SRA) database (Accession Number: SRP049145).

### Bioinformatics analysis

Pyrosequencing flowgrams were converted to sequence reads by using MOTHUR 1.15.0^38^. Trimmed sequences were produced by removing low quality sequences (quality scores <25, sequences lengths <200bp) and ambiguous reads (ambiguous base >0) using QIIME v. 1.3.0^39^. Then, sequences were denoised using DeNoiser (v0.851)^40^ and screened for chimeras using UCHIME^41^. All Archaea, Eukaryota, chloroplasts, mitochondria and unknown sequences were removed. The filtered sequences were then clustered into operational taxonomic units (OTUs) at a 3% dissimilarity level using the average neighbor method. Finally, taxonomy was assigned against the SILVA database using k-mer searching method by MOTHUR^42^.

### Statistical analysis

Chao1 richness estimator, Shannon-Wiener diversity index (H’) and Pielou evenness index (J) were calculated by MOTHUR 1.15.0^38^. The ‘niche breadth’ approach^43^,^44^ was used to identify specialization level of cyanobacteria, in which B-value was calculated by


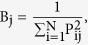


where B_j_ indicates niche breadth and P_ij_ is the relative abundance of OTU j present in a given habitat i. Principal coordinate analysis (PCoA) was conducted by using CANOCO for Windows Version 5.0 to demonstrate the dissimilarity of OTU_0.03_ compositions among different lakes. Pearson correlation analysis was carried out by SPSS version 17. Trophic status index was calculated using five variables - Chl-a (mg/m^3^), TN (mg/L), TP (mg/L), CODmn (mg/L) and SD (m) according to China National Environmental Monitoring Center (2001)^45^ and Jin (1990)^46^. Trophic status index = ∑W_j_·TLI_j_, where W_j_ is the weight of the variable j, TLI_j_ is the value of the variable j; 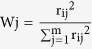 (r_ij_-the Pearson correlation between Chl-a and the variable j, m- the number of variables)(Table S2). Effects of selected environmental variables were summarized using (partial) canonical correspondence analysis (CCA) by CANOCO for Windows Version 5.0. The contributions of water physicochemical property (P), lake morpholoy (M) and watershed land-use (L) to cyanobacteria variation was evaluated using variance partitioning analysis (VPA) by CANOCO for Windows Version 4.5 (Plant Research International, Wageningen, The Netherlands). The significance test was carried out by Monte Carlo permutation (999 times). The neutral community model (NCM) proposed by Sloan *et al.*^47^,^48^ was employed to quantify the importance of neutral drivers in community assembly of cyanobacteria^47^,^48^. Model description was detailed in Liao *et al.*^43^.

## Results

### Biogeographic pattern, niche breadth and taxonomic composition of cyanobacteria

**Figure 2:**
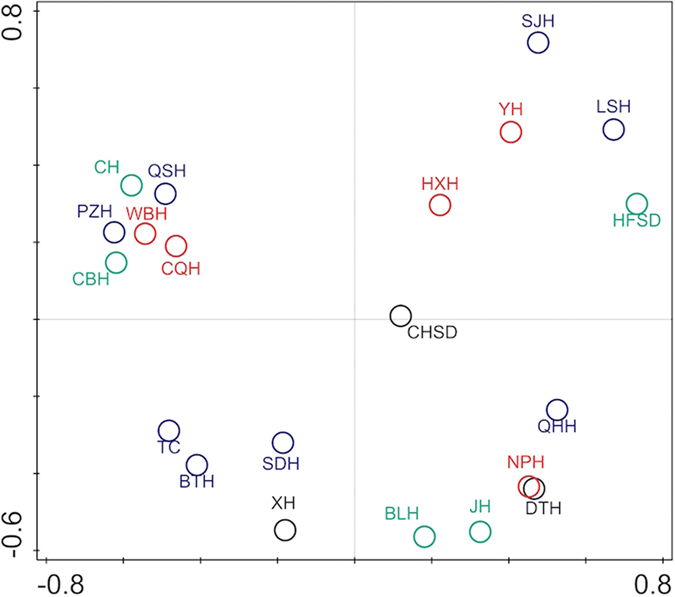
PCoA plot of cyanobacterial OTUs_0.03_. PCo Axis 1 explained 21.93% of the variation; PCo Axis 2 explained 15.46% of the variation. Black nodes represent the lakes with a Shannon-Wiener diversity (H’) > 2; red nodes represent the lakes with a H’ between 1.5 and 2; blue nodes represent the lakes with a H’ between 1 and 1.5; green nodes represent the lakes with a H’ < 1.

In total, 166,089 high quality sequences from 21 libraries of bacterial 16S rRNA genes were recovered from water samples, among which 7,455 sequences were cyanobacterial origin, which generated 94 cyanobacterial OTUs_0.03_. Results showed that DTH was inhabited by most diverse and richest cyanobacteria, followed by XH and CHSD (Table 1). SJH had the lowest richness, and BLH and CBH had the lowest diversity (Table 1). QHH and LSH, with the highest Pielou index J, exhibited the most even distribution of cyanobacteria. In the PCoA plot, CH, QSH, PZH, WBH, CBH and CQH clustered together, suggesting a similar composition of cyanobacteria (Fig. 2). Other sites exhibited a dispersed distribution. In general, these lakes showed a distinct biogeographic pattern.

**Figure 3:**
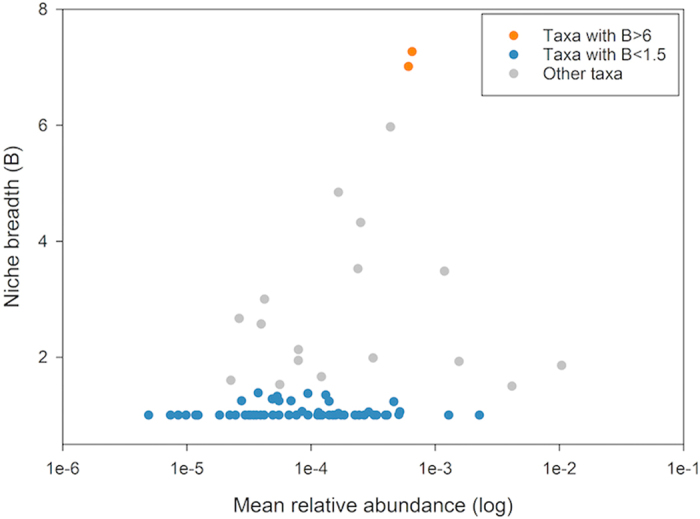
Niche breadth and mean relative abundance distribution of cyanobacteria. Each dot represents a cyanobacterial OTU_0.03_. OTUs_0.03_ with a niche breadth >6 were indicated by orange dot. OTUs_0.03_ with a niche breadth <1.5 were indicated by blue dots. Other OTUs_0.03_ were indicated by grey dots.

Niche breadth analysis revealed that most of the cyanobacteria had a low niche breadth (B-value <1.5). Only two cyanobacterial OTUs_0.03_ had a niche breadth >6 (Fig. 3). It suggested that most cyanobacterial taxa have a narrow habitat preference. A total of 10 cyanobacterial genera - *Synechococcus*, *Gloeobacter*, *Nostoc*, *Oscillatoria*, *Microcystis*, *Planktothrix*, *Phormidium*, *Leptolyngbya*, *Limnothrix*, and *Pseudanabaena* - were identified (Table S3). DTH was dominated by *Leptolyngbya* (13.64%), while HFSD was dominated by *Microcystis* (66.67%) (Table S3). In other lakes, *Synechococcus* was detected as the most prevalent genus. No significant correlation was detected between *Synechococcus* and environmental variables (Table S4).

### Key environmental factors regulating the distribution of cyanobacteria

To identify the significant environmental factors regulating the distribution of cyanobacteria, variables of physicochemical property, lake morphology and watershed land-use were analyzed in CCA (Table S1). Pearson’s correlation analyses were conducted first for variables of each category to avoid the arching effect in CCA. For water physicochemical variables, SRP had a significant correlation with TP (r = 0.685, p < 0.01) (Table S5), and had very low concentration. Thus, SRP was excluded. NO_3_-N and TKN had a significant correlation with TN (r_1_ = 0.793, r_2_ = 0.918, p < 0.01). NH_4_^+^-N was significantly correlated with TP (r = 0.699, p < 0.01) and CODmn (r = 0.621, p < 0.01) (Table S5). Thus, NO_3_-N, TKN and NH_4_^+^-N were all excluded. Salinity, pH, DO, temperature and trophic status index were selected to represent water physicochemical property. For lake morphological variables, area was the only included variable. For watershed land-use variables, forestland had a significant negative correlation with agricultural land (r = −0.921, p < 0.01), urban land (r = −0.823, p < 0.01) and barren land (r = −0.565, p < 0.01) (Table S6). Thus, forestland was excluded.

Selected variables were analyzed in CCA and results showed their considerable influence on the distribution of cyanobacteria in plateau lakes (Table 2). Among the selected physicochemical variables, DO and salinity significantly explained about 20% of the variation (p < 0.01), respectively, and trophic status index and pH explained about 15% (p < 0.05), respectively. Lake area significantly explained 18% of the variation. And among the selected land-use variables, 20%, 11% and 11% of the variation were significantly explained by urban land, water body and agricultural land, respectively (Table 2). Thus, water DO, salinity, trophic status, pH, lake area, and watershed urban land, water body and agricultural land were considered as the key environmental factors influencing the distribution of cyanobacteria in these 21 plateau lakes.

### Importance of niche and neutral processes for cyanobacterial assembly

**Figure 4:**
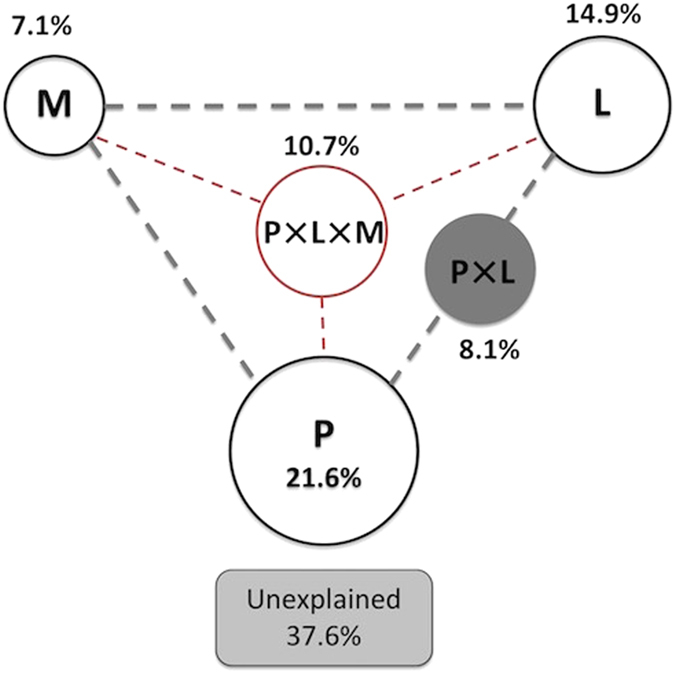
Contributions of environmental divers shaping the distribution of cyanobacteria. P-water physicochemical property, M-lake morphology, L-watershed land-use. The geometric areas were proportional to the respective percentages of explained variation.

VPA was performed to quantify the importance of niche processes by analyzing the contributions of water physicochemical property (P), lake morphology (M), and watershed land-use (L) to the cyanobacterial variation. The total variation was partitioned into the independent effects of P, M and L, interactions between any two drivers (P × M, P × L, M × L), interaction of all three drivers (P × M × L) and the unexplained part (Fig. 4). A total of 62.4% of the variation was significantly explained (p < 0.01) by these three drivers, indicating that niche process was playing an important role in cyanobacterial assembly. Water physicochemical property, lake morphology and watershed land-use independently explained 21.6%, 7.1% and 14.9% of the total variation. The interaction between water physicochemical property and watershed land-use was relatively strong (P × L, 8.1%). The combination of these three drivers explained a relatively large fraction (10.7%) of cyanobacterial variation, respectively. Thus, water physicochemical property was the most dominant diver shaping the distribution of cyanobacteria followed by watershed land-use and lake morphology.

**Figure 5:**
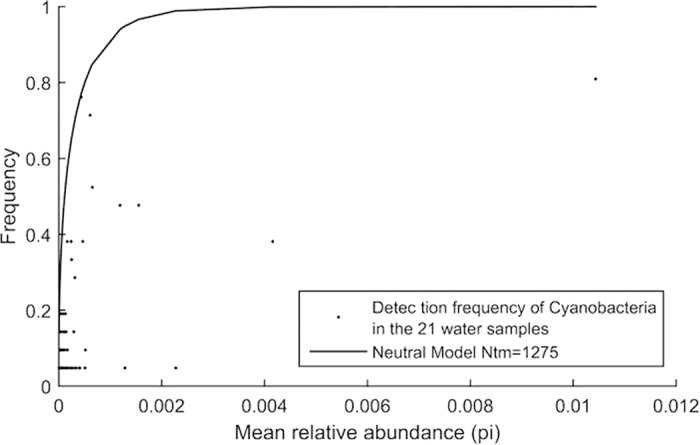
Detection frequencies of cyanobacterial OTUs_0.03_ as a function of mean relative abundance. The solid black line is the best-fit (least square error) neutral curve.

NCM was employed to evaluate the importance of neutral process in cyanobacterial assembly. The best-fit neutral curve indicated that the relationship between detection frequency and mean relative abundance (pi) of OTU_0.03_ is purely driven by neutral process. Results showed that the observed data for cyanobacteria displayed large departure from the best-fit neutral curve (Fig. 5, R^2^ = −0.51; negative R^2^ values mean that there is no fit to the model). Thus, neutral process was not at play in the assembly of cyanobacteria.

## Discussion

The diversity of cyanobacteria has been previously explored in many environmental settings such as eutrophic lake^20^, soil^6^, phyllosphere^5^, hot spring^2^,^49^ and marine habitat^50^. Here we presented the first study describing the biogeographic pattern of cyanobacteria in numerous plateau lakes, a rarely explored environment. Also, the assembly mechanism of cyanobacteria and quantified the importance of drivers shaping the biogeography of cyanobacteria have been clarified. This study would deepen the understanding of the ecology of cyanobacteria, limnology of plateau lake, and benefit the watershed management.

The 21 lakes on Yungui Plateau possessed different compositions of cyanobacteria and showed distinct biogeographic pattern (Table 1, Fig. 2). This pattern was strongly influenced by niche process rather than neutral process (Figs 4 and 5), which was consistent with Drakare and Liess (2010)’ study^26^. A total of 62.4% of the cyanobacterial variation was explained by water physicochemical property, lake morphology and watershed land-use (Fig. 4). These results indicated that cyanobacteria were not ecologically equivalent and were under strong environmental selection. They could not easily follow the random dispersal from a regional source pool of equivalently fit species. Due to the strong dependence on environmental conditions, they might undergo extinction if drastic disturbances in water physicochemical property, lake morphology and watershed land-use occur. The niche-based assembly mechanism of cyanobacteria explains their narrow habitat preference indicated by low niche breadth (Fig. 3), as previous study has demonstrated that specialists are assembled via niche processes rather than neutral processes^43^. Although a wide range of environmental variables were included in the present study, 34.24% of the variation still could not be explained (Fig. 4). This might result from the incompleteness of the environmental index setting, e.g. daylight duration, precipitation, climate change, water column stability, zooplankton abundance, etc.^51^. Moreover, using general bacterial primers instead of specific primers for cyanobacteria might lead to a loss of explained variation. Among all the lakes, DTH exhibited the richest and most diverse cyanobacteria (Table 1). This could be explained by its environment condition with high salinity, high DO, rich nutrient, large area and intense disturbance of agriculture and urbanization (Table S1).

Water physicochemical property was the most dominant driver in shaping the distribution of cyanobacteria, which was consistent with the results of Özkan *et al.*^25^ and Drakare and Liess^26^. Significant physicochemical factors were identified as DO, salinity, trophic status and pH (Table 2). The coherent relationship between DO and bacterial community had been detected in various freshwater environments^52^,^53^,^54^. It indicated that the bacterial community could be strongly driven by electron acceptor availability, inducing probably different metabolic pathways^52^. Salinity was identified in many studies as a critical factor influencing cyanobacteria taxa^2^,^5^,^6^. The effects of salinity on microbial cells were related to osmoregulation and/or to metabolic changes triggered by salt, such as the ability to uptake different dissolved organic carbon compounds^55^. It was reported that cyanobacteria had the ability to tolerate high salinity^56^. Trophic status has been shown to influence cyanobacteria in numerous studies^9^,^8^,^57^,^58^. In other words, the richness and diversity of cyanobacteria could well reflect the trophic status, and thus could be used as an indicator of eutrophication for water management in plateau lakes. As nitrogen and phosphorus are limiting factors for the growth and productivity of cyanobacteria, high loading of nitrogen and phosphorus could lead to high cyanobacterial population^9^,^25^. Organic pollution, represented by CODmn, might affect cyanobacteria by providing carbon resource, as cyanobacteria could grow heterotrophically under stress conditions^8^. pH could influence the activity of enzyme in microbial cells and further influence the metabolism of cyanobacteria^25^.

Watershed land-use, especially urban land, water body and agricultural land strongly influenced the distribution of cyanobacteria independently or combined with other environmental drivers (Fig. 4). Recent study by Machmuller *et al.*^59^ proved that emerging land use practices rapidly increased soil organic matter^59^. The increased organic matter in soil could be further transferred by groundwater flows to other water bodies such as inflowing rivers, and finally reached the lakes. Agricultural land and urban land were also recognized as the main sources of nitrogen and phosphorus input in lakes^24^. The input of organic matter, nitrogen and phosphorus would further change biochemical features of lake and alter the growth rate and yield of cyanobacteria. However, in Özkan *et al.*’s^25^ study, land-use in catchments contributed little to the variation of cyanobacterial diversity^25^. The difference in the importance of land-use might be attributed to the different location and natural conditions of habitats. Due to the influence of subtropical plateau monsoon climate and landform of Yungui Plateau, China, lakes studied in the present study had relatively lower water supply, rate of water exchange and resilience compared to the lakes in plains. The ecosystems of these plateau lakes thus showed relative higher vulnerability to human disturbance^28^,^31^. As a result, cyanobacteria in plateau lakes might be more sensitive to the changes caused by land-use practice, which explained why watershed land-use here acted as a critical driver in regulating cyanobacterial distribution, rather than in freshwater habitats in Özkan *et al.* (2013)’s study^25^.

Lake morphology represented by area was the least important driver. The possibility that the lake area could influence cyanobacteria might be that lakes with larger area could provide larger habitats and more water resource for cyanobacteria^25^. Thus, larger lake areas would tend to harbor greater cyanobacterial richness, suggesting a species-area relationship^60^, which has been widely established for both macrobes and microbes^61^.

Most of the genera identified in the present study were known to contain toxin-producing strains: *Nostoc*^11^, *Oscillatoria*^11^,^13^, *Microcystis*, *Planktothrix*^11^,^62^, *Phormidium*^63^,^64^,^65^, *Leptolyngbya*, *Limnothrix*, *Pseudanabaena*^13^. *Synechococcus*, the most abundant cyanobacterial genus in these 21 plateau lakes, was also thought to have a potential of high cytotoxic activity^66^, in spite of its significant role in the nitrogen and carbon inputs in different ecosystems^6^,^67^. Toxic producer, not necessarily being the dominant species but just existing in smaller amounts, could produce large amounts of toxin in lakes^1^. Moreover, it was reported that if more than one microcystin-producing genus exists in a lake, any one of them could potentially become dominant or to form blooms in response to changed conditions^68^. Thus, these lakes on Yungui Plateau have high possibility to encounter toxicity pollution. Nevertheless, further analyses for toxin production by these potential toxin-producers are needed to confirm the risk of toxin pollution. Rantala *et al.*^68^ showed that the occurrence probability of microcystin synthetase gene E (*mcyE*) could be predicted by total nitrogen, pH, and the surface area of the lake, whereas total phosphorus alone was responsible for microcystin concentrations^68^. Meanwhile considering the importance of environmental factors demonstrated in the present study, it suggested that efforts to reduce nutrient loading, and control pH and surface area could be efficient in lowering the occurrence probability of toxic cyanobacterial genera and minimizing the risk of toxin pollution. In addition, considering the significant impact of watershed land-use, reducing land-use practices could be beneficial to prevent eutrophication and cyanobacteria bloom.

## Additional Information

**How to cite this article**: Liao, J. *et al.* Cyanobacteria in lakes on Yungui Plateau, China are assembled via niche processes driven by water physicochemical property, lake morphology and watershed land-use. *Sci. Rep.* **6**, 36357; doi: 10.1038/srep36357 (2016).

**Publisher’s note:** Springer Nature remains neutral with regard to jurisdictional claims in published maps and institutional affiliations.

## Supplementary Material

Supplementary Information

## Figures and Tables

**Table 1:** Estimation of the cyanobacterial diversity based on 16S rRNA gene pyrosequencing in 21 plateau lakes.

	No. of observed OTU_0.03_	Chao1_0.03_	Shannon-Wiener diversity index H’	Pielou evenness index J
DTH	55	55	2.99	0.75
YH	7	17	1.69	0.87
BLH	14	14	0.40	0.15
QHH	3	6	1.10	1.00
CQH	8	11	1.64	0.79
CH	6	7	0.72	0.40
XH	28	29	2.78	0.84
NPH	8	8	1.83	0.88
CHSD	20	29	2.52	0.84
LSH	4	10	1.39	1.00
HFSD	3	4	0.87	0.79
PZH	6	6	1.03	0.58
SJH	3	3	0.96	0.87
SDH	15	15	1.43	0.53
WBH	11	12	1.69	0.70
JH	11	14	0.69	0.29
TC	12	13	1.26	0.51
CBH	5	7	0.40	0.25
BTH	10	11	1.37	0.60
HXH	7	8	1.71	0.88
QSH	8	18	1.13	0.54

**Table 2:** Effects of selected environmental variables in CCA.

Variables	Explains %	pseudo-F	P
Water physicochemical variables			
DO	20.4	4.9	**0.004**
Salinity	20.2	4.8	**0.008**
Eutrophication index	15.8	3.6	**0.014**
pH	15.1	3.4	**0.008**
Temperature	6.30	1.3	0.2
Lake morphological variable			
Area	17.7	4.1	**0.004**
Watershed land-use variables			
Urban land	19.6	4.6	**0.014**
Water body	10.8	2.3	**0.012**
Agricultural land	10.8	2.3	**0.026**
Grassland	7.90	1.6	0.126
Barren land	7.70	1.6	0.074
