# Supplementary material for: Cyanobacteria in lakes on Yungui Plateau, China are assembled via niche processes driven by water physicochemical property, lake morphology and watershed land-use
Source: Sci Rep. 2016 Nov 7;6:36357. doi: 10.1038/srep36357 (PMC5098255; doi:10.1038/srep36357)
Supplement: Supplementary Information [file srep36357-s1.pdf]

Supplementary information

Cyanobacteria in lakes on Yungui Plateau, China are assembled via niche processes driven by water physicochemical property, lake morphology and watershed land-use Running title: Assembly mechanism of cyanobacteira

Jingqiu LIAO[1#], Lei ZHAO[2], Xiaofeng CAO[1], Jinhua SUN[3], Zhe GAO[1], Jie WANG[1], Dalin JIANG[1], Hao FAN[1], Yi HUANG[1*]

1 State Key Joint Laboratory of Environmental Simulation and Pollution Control, College of Environmental Sciences and Engineering, Peking University, Beijing, 100871, China

2 Yunnan Key Laboratory of Pollution Process and Management of Plateau Lake – Watershed, Kunming, 650034, China

3 Chinese Research Academy of Environmental Sciences, Beijing, 100012, China

[#] Present address: Department of Microbiology, Cornell University, Ithaca, NY 14853, USA

*Corresponding Author: College of Environmental Sciences and Engineering, Peking University, No. 5 Yiheyuan Road, Haidian District, Beijing, People's Republic of China 100871

E-mail: yhuang@pku.edu.cn

Fax number: +86 10 62757867

Table S1 The environmental variables data of the water samples from 21 plateau lakes

	DTH	YH	BLH	QHH	CQH	CH	XH	NPH	CHSD	LSH	HFSD	PZH	SJH	SDH	WBH	JH	TC	CBH	BTH	HXH	QSH
Water physicochemical variables																					
Chl-a (mg/m^3)	29.18	8.10	7.37	2.67	8.61	6.72	3.11	1.68	1.69	1.23	10.60	10.33	18.83	4.75	0.11	1.94	0.05	0.40	11.68	1.00	1.01
NH_4^+-N (mg/L)	0.06	0.10	0.02	0.29	0.06	0.02	0.32	0.45	0.02	0.05	0.14	0.15	0.08	0.21	0.03	0.12	0.09	0.01	0.05	0.01	0.01
NO_3-N (mg/L)	0.25	0.06	0.55	0.13	1.53	0.14	0.01	0.18	0.02	0.04	0.08	0.09	0.04	0.01	0.01	0.02	0.02	0.01	0.03	0.01	0.81
TKN (mg/L)	0.31	0.12	0.58	0.41	1.61	0.18	0.32	0.96	0.03	0.07	0.22	0.30	0.10	0.23	0.04	0.09	0.07	0.03	0.07	0.03	0.83
TN (mg/L)	1.14	0.20	0.65	0.97	2.00	0.21	0.49	1.24	0.06	0.11	0.28	0.63	0.47	0.29	0.05	0.24	0.19	0.03	0.09	0.03	0.87
SRP (mg/L)	0.02	0.01	0.00	0.02	0.01	0.00	0.04	0.02	0.01	0.01	0.01	0.00	0.00	0.01	0.01	0.01	0.01	0.00	0.02	0.00	0.01
TP (mg/L)	0.10	0.03	0.01	0.12	0.02	0.01	0.07	0.11	0.01	0.02	0.02	0.04	0.04	0.02	0.02	0.05	0.03	0.01	0.07	0.01	0.02
CODmn (mg/L)	8.00	3.58	1.20	9.27	5.88	4.35	8.69	5.49	2.54	4.98	5.03	6.91	5.96	5.67	3.62	4.40	3.99	2.30	4.17	2.98	1.72
Trophic status index	61.91	44.43	39.65	39.63	49.26	41.50	42.13	37.99	29.98	30.09	47.72	50.67	55.28	41.37	10.86	36.19	6.56	16.17	47.87	23.91	27.24
Salinity (‰)	326.26	70.55	105.13	257.57	222.03	90.83	217.07	113.12	105.22	96.50	82.32	94.54	181.22	22.28	84.55	135.23	39.06	133.83	38.37	95.28	52.65
pH	7.32	8.89	8.49	6.97	9.41	9.34	7.72	7.55	8.32	9.44	10.11	8.85	7.26	7.57	9.06	8.22	8.01	8.43	7.84	8.76	8.99
DO (mg/L)	13.40	2.69	7.50	6.97	8.13	8.19	2.03	3.90	8.59	9.60	8.37	8.51	7.30	5.19	6.47	6.07	5.77	6.54	6.31	7.08	8.01
Temperature (°C)	22.03	24.00	24.57	23.27	28.67	18.37	22.00	25.40	27.87	14.57	20.53	21.67	26.07	19.33	21.60	21.00	16.63	14.93	15.67	19.23	21.90
SD (m)	0.37	2.00	3.50	9.94	0.98	2.77	1.28	1.15	1.43	2.00	1.78	1.00	0.50	2.33	1.32	0.79	1.92	3.65	1.60	2.90	2.83
Lake morphologic variable																					
Area (km^2)	10.98	0.90	3.24	3.41	10.24	0.90	3.27	14.98	0.61	7.62	1.99	5.50	2.30	1.44	2.20	4.81	1.16	8.39	1.69	3.84	5.21
Watershed land-use variables																					
Agricultural land (%)	45	35	42	41	58	9	28	4	25	26	29	52	51	0	6	18	0	24	0	16	36
Forest land (%)	16	51	42	43	11	72	50	68	45	64	63	27	21	62	76	69	89	51	87	67	44
Grass land (%)	10	3	1	4	3	1	15	18	15	3	2	2	9	29	13	9	3	18	3	13	10
Water body (%)	7	2	2	3	8	18	2	1	1	5	2	4	3	8	5	1	8	3	9	2	5
Urban land (%)	15	2	2	8	17	0	5	3	4	2	1	3	8	0	0	2	0	4	0	1	3
Barren land (%)	6	7	11	1	3	0	1	6	11	1	4	12	9	1	0	1	0	0	1	0	2
Lake geographic location																					
Longitude (E)	103.31	103.45	103.99	100.60	103.36	103.71	100.04	99.64	100.24	100.14	103.63	104.09	103.30	99.95	100.19	99.93	99.28	99.94	99.99	99.96	103.11
Latitude (N)	23.43	24.84	24.14	25.44	23.44	24.58	26.02	27.88	26.23	26.88	25.75	24.16	23.59	27.91	27.06	26.49	25.87	26.16	27.82	26.28	25.60
Altitude (m)	1280	1908	1494	1975	1283	1889	1965	3266	2192	2438	1954	1445	1273	3612	3078	2186	2555	2050	3541	2130	2172

Table S2 Pearson's correlation matrix of eutrophication index

	Chl-a	TP	TN	SD	CODmn
Chl-a	1.000				
TP	0.302	1.000			.
TN	0.313	0.408	1.000	.	
SD	-0.323	0.204	0.032	1.000	
CODmn	0.386	0.702**	0.412	0.126	1.000

**. Significantly correlated at 0.01 level(bilateral)

Table S3 Freshwater cyanobacteria identified at genus level in the 21 plateau lakes

Taxon*	*Synechococcus*	*Gloeobacter*	*Oscillatoria*	*Planktothrix*	*Microcystis*	*Phormidium*	*Leptolyngbya*	*Nostoc*	*Pseudanabaena*	*Limnothrix*
DTH	0.00	0.32	3.47	0.00	0.00	2.12	13.64	8.71	0.42	0.00
YH	23.08	0.00	0.00	0.00	0.00	0.00	0.00	0.00	0.00	0.00
BLH	97.06	0.00	0.00	0.00	0.37	0.00	0.00	0.00	0.00	0.00
QHH	33.33	0.00	0.00	0.00	33.33	0.00	0.00	0.00	0.00	0.00
CQH	73.13	0.00	0.00	0.00	1.49	0.00	0.00	0.00	0.00	0.00
CH	87.80	0.00	0.00	0.00	0.00	2.44	0.00	0.00	0.00	0.00
XH	30.28	0.00	0.00	8.14	2.04	0.00	0.00	0.00	0.51	1.02
NPH	0.00	0.00	0.00	0.00	0.00	0.00	0.00	0.00	0.00	0.00
CHSD	12.50	0.00	0.00	0.00	0.00	0.00	0.00	0.00	0.00	0.00
LSH	25.00	0.00	0.00	0.00	0.00	0.00	0.00	0.00	0.00	0.00
HFSD	0.00	0.00	0.00	0.00	66.67	0.00	0.00	0.00	0.00	0.00
PZH	92.73	0.00	0.00	0.00	0.00	0.00	0.00	0.00	0.00	0.00
SJH	57.14	0.00	0.00	0.00	0.00	0.00	0.00	0.00	0.00	0.00
SDH	82.33	0.00	0.00	0.00	0.00	0.00	0.00	0.00	0.00	0.00
WBH	65.57	0.00	0.00	0.00	0.00	0.00	1.64	0.00	0.00	0.00
JH	2.96	0.00	0.00	0.00	0.23	0.00	0.00	0.00	0.00	0.00
TC	79.90	0.00	0.00	0.00	0.00	0.00	0.00	0.00	0.00	0.00
CBH	93.22	0.00	0.00	0.00	0.00	0.00	0.00	0.00	0.00	0.00
BTH	50.68	0.00	0.00	0.00	0.00	0.00	0.00	0.00	0.00	0.00
HXH	51.72	0.00	0.00	0.00	10.34	0.00	0.00	0.00	0.00	0.00
QSH	70.59	0.00	0.00	0.00	1.96	0.00	0.00	0.00	0.00	0.00

* Percent of total number of sequence reads with cyanobaterial origin

Table S4 Pearson's correlation matrix of *Synechococcus* and abiotic environmental variables

	Synechococcus
Chl-a	0.004
NH3	-0.151
NO3	0.206
TKN	0.143
TN	0.045
PO4	-0.222
TP	-0.239
CODmn	-0.366
Salinity	-0.115
pH	-0.017
DO	-0.015
Temperature	0.120
SD	0.135
Area	-0.128
Agricultural land	0.119
Forest land	-0.061
Grass land	-0.177
Water body	-0.067
Urban land	-0.136
Barren land	0.334

Table S5 Pearson's correlation matrix of physicochemical variables

	Chl-a	NH_4^+-N	NO_3-N	TKN	TN	SRP	TP	CODmn	Salinity	pH	DO	Temperature	SD
Chl-a	1.000												
NH_4^+-N	-0.118	1.000											
NO_3-N	0.101	-0.173	1.000										
TKN	0.044	0.255	0.896**	1.000									
TN	0.313	0.328	0.793**	0.918**	1.000								
SRP	0.084	0.655**	-0.109	0.136	0.233	1.000							
TP	0.302	0.699**	-0.151	0.151	0.408	0.685**	1.000						
CODmn	0.386	0.621**	-0.078	0.133	0.412	0.633**	0.702**	1.000					
Salinity	0.494*	0.195	0.243	0.286	0.575**	0.406	0.541*	0.651**	1.000				
pH	-0.227	-0.469*	0.282	0.092	-0.142	-0.386	-0.663**	-0.442*	-0.393	1.000			
DO	0.512*	-0.521*	0.233	0.029	0.194	-0.302	-0.058	0.024	0.330	0.180	1.000		
Temperature	0.212	0.219	0.470*	0.555**	0.596**	0.099	0.157	0.139	0.391	-0.135	-0.032	1.000	
SD	-0.323	0.154	-0.031	-0.004	0.032	-0.064	0.204	0.126	0.142	-0.209	-0.058	-0.098	1.000

**. Significantly correlated at 0.01 level(bilateral)

*. Significantly correlated at 0.05 level(bilateral)

Table S6 Pearson's correlation matrix of anthropogenic geographic variables

	Agricultural land	Forest land	Grass land	Water body	Urban land	Barren land
Agricultural land	1.000					
Forest land	-0.921**	1.000				
Grass land	-0.389	0.083	1.000			
Water body	-0.251	0.172	-0.219	1.000		
Urban land	0.710**	-0.823**	-0.087	-0.002	1.000	
Barren land	0.549*	-0.565**	-0.187	-0.360	0.206	1.000

**. Significantly correlated at 0.01 level(bilateral)

*. Significantly correlated at 0.05 level(bilateral)
